# Machine learning–based prediction of in-hospital deep vein thrombosis in patients with acute ischemic stroke: a multicenter study

**DOI:** 10.3389/fneur.2026.1847449

**Published:** 2026-08-14

**Authors:** Tieshi Zhu, Runzhui Lin, Le Zhao, He Zhu

**Affiliations:** 1 Department of Neurology, Zhanjiang Central Hospital, Guangdong Medical University, Zhanjiang, Guangdong, China; 2 Department of Hepatobiliary, Pancreatic, and Splenic Surgery, Second Affiliated Hospital of Shantou University Medical College, Shantou, China; 3 Department of Medical Affairs, Central Hospital of Guangdong Provincial Nongken, Zhanjiang, Guangdong, China; 4 Department of Clinical Research Institute, Zhanjiang Central Hospital, Guangdong Medical University, Zhanjiang, China

**Keywords:** acute ischemic stroke, deep vein thrombosis, machine learning, online calculator, risk prediction, thromboprophylaxis

## Abstract

**Background:**

Deep vein thrombosis (DVT) is a common complication of acute ischemic stroke (AIS) and may worsen clinical outcomes, yet reliable tools for early risk stratification remain limited. We aimed to develop and internally evaluate machine learning models for predicting in-hospital DVT in patients with AIS.

**Methods:**

We conducted a secondary analysis of a publicly available multicenter retrospective dataset including 21,459 patients with AIS. The primary outcome was imaging-confirmed in-hospital DVT. Participants were stratified according to DVT status and randomly divided into a training set (70%) and a held-out test set (30%). Feature selection was performed using least absolute shrinkage and selection operator regression, the Boruta algorithm, variance inflation factor assessment, and clinical judgment. Eight machine learning algorithms were trained using a 19-variable full predictor set and an 8-variable simplified predictor set. Hyperparameters were optimized using repeated 5-fold cross-validation with 2 repeats. Model performance was evaluated using the area under the receiver operating characteristic curve (AUC), area under the precision-recall curve (AUPRC), Brier score, calibration, decision curve analysis, and additional classification metrics. Sensitivity analyses excluded D-dimer and used within-fold synthetic minority oversampling.

**Results:**

Among 21,459 patients, 1,324 (6.17%) developed in-hospital DVT. In the full predictor-set analysis, RANGER achieved the highest AUC in the held-out test set (0.976). Among models using the simplified predictor set, XGBoost achieved the highest AUC (0.917) and sensitivity (0.852), whereas SVM demonstrated the most favorable overall performance profile, with the highest AUPRC (0.605), lowest Brier score (0.038), highest positive predictive value (0.440), and highest *F*1-score (0.549). D-dimer was the most influential predictor. Model performance was attenuated after exclusion of D-dimer, and the SMOTE analysis showed slightly lower discrimination and greater calibration discrepancies. All 8 simplified predictor-set models were incorporated into an online prediction platform.

**Conclusion:**

Machine learning models demonstrated favorable performance for predicting in-hospital DVT after AIS. Among models using the simplified 8-variable predictor set, SVM showed the most favorable overall performance, whereas XGBoost prioritized sensitivity. Independent external validation and prospective clinical-impact assessment are required before routine clinical implementation.

## Introduction

Acute ischemic stroke (AIS) remains one of the leading causes of disability and mortality worldwide, and a substantial proportion of patients experience suboptimal functional recovery during hospitalization (1–3). Among the various complications, deep vein thrombosis (DVT) is particularly common and clinically significant (4–6). In China, the reported incidence of DVT after acute stroke ranges from 8.9 to 28% (7). The occurrence of DVT may not only progress to life-threatening pulmonary embolism but can also delay early mobilization and rehabilitation, thereby further worsening functional outcomes (8, 9). Despite this, routine anticoagulant prophylaxis is not universally applied in patients with AIS (10). Concerns regarding bleeding risk—especially in the context of widespread antiplatelet therapy—as well as the absence of reliable risk stratification tools, often limit its use in clinical practice (11). Therefore, identifying patients at high risk of DVT and implementing targeted preventive strategies remain critical unmet needs.

Previous studies have explored risk factors for DVT in patients with AIS; however, most were based on conventional regression models with limited sample sizes and were often conducted in single-center settings (12, 13). Such models generally rely on prespecified functional forms and may have limited ability to capture nonlinear associations and complex interactions among clinical variables. In addition, class imbalance, calibration, clinical utility, and external validity were not consistently evaluated, and few existing models have been translated into readily accessible clinical tools (14, 15). With the increasing availability of large-scale clinical data, machine learning provides a flexible framework for modeling nonlinear relationships, integrating multidimensional predictors, and comparing alternative algorithms under a unified validation strategy (16, 17). Therefore, a systematic comparison of multiple machine learning approaches may help identify models that achieve an appropriate balance among discrimination, calibration, interpretability, and clinical feasibility.

In this study, we developed and internally evaluated a machine learning–based model to predict in-hospital DVT in patients with AIS using a large multicenter retrospective cohort of more than 20,000 individuals. We applied a structured feature selection strategy and incorporated clinically accessible variables to enhance model interpretability and feasibility. To further facilitate clinical implementation, we developed a simplified predictor set, retrained all 8 algorithms, and incorporated the resulting models into an online prediction platform for further evaluation. The models were intended to support research on early risk stratification rather than immediate clinical decision-making.

## Methods

### Reporting guideline

This study was reported in accordance with the TRIPOD + AI guideline for studies developing and evaluating clinical prediction models using regression or machine learning methods. A completed TRIPOD + AI checklist is provided in Supplementary material.

### Study population

This study was a secondary analysis of a publicly available deidentified dataset deposited in the Dryad Digital Repository (DOI: 10.5061/dryad.w0vt4b92c) and derived from a multicenter retrospective cohort study (18). The original cohort was established at 4 hospitals in Chongqing, China, including Chongqing Emergency Medical Center (Chongqing University Central Hospital), Qianjiang Central Hospital, Bishan District People’s Hospital, and Yubei District Traditional Chinese Medicine Hospital, and consecutively enrolled patients with AIS between June 2017 and July 2023. However, nationality and ethnicity were not recorded in the publicly available dataset. The eligibility criteria for the present analysis followed those of the source cohort. Briefly, eligible participants were adults aged 18 to 90 years with a confirmed diagnosis of AIS. The source cohort excluded patients with a history of stroke or transient ischemic attack, pre-existing neurologic disorders, including traumatic brain injury, intracranial tumors, or cerebrovascular malformations, prior epilepsy or anti-seizure medication use, or death within 72 h after stroke onset. The publicly released analytic dataset included 21,459 eligible patients, all of whom were included in the present analysis. Because records with incomplete outcome or candidate predictor data had already been excluded during preparation of the source dataset, no additional missing-data imputation was performed. Information on the number and characteristics of patients excluded because of missing data was not available in the public dataset.

### Outcome

The primary outcome was in-hospital DVT, defined as thrombus formation in the deep venous system confirmed during the index hospitalization by compression ultrasonography or other imaging modalities according to routine clinical practice. Candidate predictors were restricted to variables described as baseline or admission measurements in the source dataset. However, exact timestamps of individual laboratory measurements relative to DVT diagnosis were not available in the publicly released dataset.

### Candidate variables

Candidate predictors were pre-specified from variables described as being obtained at admission or during the initial clinical assessment in the source dataset. These included demographic characteristics, baseline stroke-related features, vascular risk factors and comorbidities, and laboratory parameters. Because exact measurement timestamps were unavailable, the temporal relationship between individual laboratory measurements and DVT diagnosis could not be independently verified. Demographic and baseline clinical variables included age, sex, and stroke severity assessed using the National Institutes of Health Stroke Scale (NIHSS). Stroke-related variables included infarction location (e.g., subcortical infarction and posterior circulation involvement) and etiologic subtype (e.g., large artery atherosclerosis). Comorbidities included fatty liver disease, diabetes mellitus, hypertension, coronary artery disease, atrial fibrillation, hyperuricemia, hyperlipidemia, and hypoproteinemia. Laboratory variables included hematologic indices, inflammatory and metabolic markers, liver and renal function parameters, coagulation markers, cardiac-related biomarkers, and electrolyte/metabolic indices. Variables that could represent postadmission complications or events occurring after the baseline assessment were not considered candidate predictors for model development. Detailed information on preadmission antiplatelet or anticoagulant therapy, as well as in-hospital pharmacologic or mechanical thromboprophylaxis, was not available in the publicly released dataset and therefore could not be incorporated into model development.

### Feature selection

Feature selection was performed exclusively in the training set to minimize information leakage. We first applied least absolute shrinkage and selection operator (LASSO) regression and the Boruta algorithm to identify candidate predictors associated with in-hospital DVT. Variables retained by either method were then further evaluated for multicollinearity using variance inflation factors (VIFs) derived from a multivariable logistic regression model. A VIF greater than 10 was considered indicative of severe multicollinearity. Variables were subsequently evaluated according to redundancy, clinical interpretability, predictive contribution, and availability in routine practice. The remaining variables were subsequently reviewed in light of clinical interpretability and availability in routine practice. Through this stepwise process, 19 variables were selected to form the full predictor set: hypertension, atrial fibrillation, age, C-reactive protein, triglycerides, serum albumin, blood urea, activated partial thromboplastin time, thrombin time, international normalized ratio, D-dimer, α-hydroxybutyrate dehydrogenase, ischemia-modified albumin, serum chloride, serum phosphorus, lactate, anion gap, total carbon dioxide, and NIHSS score.

To improve clinical usability, we subsequently developed a simplified predictor set. Predictor reduction was based on Shapley additive explanations (SHAP)-derived feature importance obtained from the full RANGER model fitted in the training data, together with routine clinical availability, ease and timeliness of measurement, interpretability, redundancy, measurement burden, and feasibility for online implementation. The held-out test set was not used during predictor reduction. Eight variables were ultimately retained in the simplified predictor set: age, atrial fibrillation, D-dimer, NIHSS score, C-reactive protein, serum albumin, lactate, and blood urea. All 8 machine learning algorithms were subsequently retrained using this simplified predictor set.

### Model development and evaluation

The dataset was randomly divided into a training set comprising 70% of participants and a held-out test set comprising the remaining 30%, with stratification according to DVT status to preserve the outcome prevalence in both datasets. Predictor selection and model development were conducted using the training set only. The held-out test set was not used for predictor selection, preprocessing decisions, hyperparameter optimization, or model comparison.

Eight prediction algorithms were evaluated using both the full and simplified predictor sets. These comprised conventional logistic regression as a benchmark and 7 machine-learning approaches: gradient boosting machine, elastic net logistic regression, random forest using the RANGER implementation, support vector machine (SVM) with a radial basis function kernel, extreme gradient boosting (XGBoost), naive Bayes, and a single-hidden-layer feed-forward neural network. Zero- and near-zero-variance predictors were removed for all models. Centering and scaling were additionally applied for elastic net logistic regression, SVM, naive Bayes, and neural network models, whereas conventional logistic regression and tree-based models were fitted without centering or scaling.

Model-specific hyperparameters were optimized in the training set using grid search with stratified repeated 5-fold cross-validation with 2 repeats. The mean area under the receiver operating characteristic curve (ROC AUC) across resampling iterations was used as the optimization criterion. Conventional logistic regression did not require hyperparameter tuning. After hyperparameter selection, each final model was fitted using the complete training set and evaluated in the held-out test set. In the primary analysis, no over-sampling or under-sampling was performed, and the original outcome distribution was retained.

Model discrimination was evaluated using ROC AUC and the area under the precision-recall curve (AUPRC). Calibration was assessed using calibration plots and the Brier score, and potential clinical utility was assessed using decision curve analysis. Additional threshold-dependent performance measures included accuracy, sensitivity (recall), specificity, positive predictive value (precision), negative predictive value, and *F*1-score. Although ROC AUC was used for hyperparameter optimization, final model comparisons considered discrimination, calibration, and metrics informative for an imbalanced outcome, with particular emphasis on AUPRC, positive predictive value, sensitivity, *F*1-score, and Brier score rather than ROC AUC alone. The optimal classification threshold was determined in the training set using the Youden index and was subsequently applied without modification to the held-out test set.

SHAP was used to quantify global predictor importance and visualize patient-level feature contributions. SHAP-derived importance from the full RANGER model was considered together with clinical availability, interpretability, redundancy, measurement burden, and implementation feasibility when deriving the simplified predictor set. XGBoost was subsequently used as a representative nonlinear tree-based model for detailed SHAP visualization. All eight simplified predictor-set models were incorporated into the online prediction platform, which provides individualized predicted probabilities and model-specific explanatory output where available.

All analyses were conducted using R version 4.3.5. Model-specific implementation, preprocessing procedures, and candidate hyperparameter search spaces are presented in Supplementary Table S1. The hyperparameters selected for the simplified predictor-set models in the primary and SMOTE sensitivity analyses are presented in Supplementary Table S2.

### Sensitivity analyses

Three sets of sensitivity analyses were performed to evaluate the robustness of the findings. First, given the dominant predictive contribution of D-dimer and the uncertainty regarding its measurement timing relative to DVT diagnosis, D-dimer was excluded from the 19-variable full predictor set, resulting in an 18-variable predictor set. All eight machine learning algorithms were then retrained and evaluated. Second, D-dimer was excluded from the 8-variable simplified predictor set, resulting in a 7-variable predictor set, and the same eight algorithms were retrained and evaluated. Third, to address class imbalance, the synthetic minority oversampling technique (SMOTE) was applied in analyses using the simplified 8-variable predictor set. SMOTE was implemented exclusively within the training portion of each cross-validation iteration. The corresponding assessment folds and the held-out test set were not resampled and retained the original DVT prevalence. All sensitivity analyses used the same data partitioning, repeated 5-fold cross-validation framework, and performance evaluation procedures as the primary analysis. The same hyperparameter search spaces were used where computationally feasible; in the SMOTE analysis, a linear-kernel SVM with candidate C values of 0.01, 0.1, and 1 was used to reduce computational burden.

## Results

### Baseline characteristics

A total of 21,459 patients with AIS were included in the analysis, including 10,843 women (50.5%). The median age was 67 years (IQR, 59–76 years), and the median NIHSS score at admission was 8 (IQR, 6–10). Overall, 5,505 patients (25.7%) had large artery atherosclerosis, 4,501 (21.0%) had posterior circulation involvement, and 2,443 (11.4%) had subcortical infarction. During hospitalization, 1,324 patients (6.17%) developed DVT (Table 1).

**Table 1:** Baseline characteristics of the train and held-out test set sets.

Variables	All (*n* = 21,459)	Test (*n* = 6,437)	Train (*n* = 15,022)	*p*
Age (year)	67.00 (59.00, 76.00)	67.00 (59.00, 76.00)	67.00 (59.00, 76.00)	0.10
Female	10,843 (50.53%)	3,241 (50.35%)	7,602 (50.61%)	0.77
NIHSS	8.00 (6.00, 10.00)	8.00 (6.00, 10.00)	8.00 (6.00, 10.00)	0.76
DVT	1,324 (6.17%)	397 (6.17%)	927 (6.17%)	>0.99
Subcortical	2,443 (11.38%)	759 (11.79%)	1,684 (11.21%)	0.23
Posterior	4,501 (20.97%)	1,309 (20.34%)	3,192 (21.25%)	0.14
LAA	5,505 (25.65%)	1,612 (25.04%)	3,893 (25.92%)	0.19
FLD	4,245 (19.78%)	1,277 (19.84%)	2,968 (19.76%)	0.91
DM	7,322 (34.12%)	2,237 (34.75%)	5,085 (33.85%)	0.21
HTN	14,751 (68.74%)	4,419 (68.65%)	10,332 (68.78%)	0.86
CAD	9,672 (45.07%)	2,901 (45.07%)	6,771 (45.07%)	>0.99
AF	2043 (9.52%)	583 (9.06%)	1,460 (9.72%)	0.14
Herniation	181 (0.84%)	58 (0.90%)	123 (0.82%)	0.60
Hydro	281 (1.31%)	89 (1.38%)	192 (1.28%)	0.58
HUA	2,347 (10.94%)	666 (10.35%)	1,681 (11.19%)	0.07
HLP	4,469 (20.83%)	1,330 (20.66%)	3,139 (20.90%)	0.71
HypoAlb	2,421 (11.28%)	699 (10.86%)	1722 (11.46%)	0.21
PLT (10^9^/L)	187.40 (174.30, 203.20)	187.60 (174.00, 203.50)	187.40 (174.60, 203.10)	0.83
WBC (10^9^/L)	8.20 (7.60, 9.00)	8.20 (7.60, 9.10)	8.10 (7.60, 9.00)	0.01
RBC (10^9^/L)	4.30 (4.10, 4.50)	4.30 (4.10, 4.50)	4.30 (4.10, 4.50)	0.83
HbA1c (%)	6.40 (6.00, 7.20)	6.40 (6.00, 7.20)	6.40 (6.00, 7.20)	0.28
CRP (mg/dL)	9.40 (4.70, 19.10)	9.60 (4.80, 19.60)	9.25 (4.70, 18.80)	0.03
TG (mmol/L)	1.50 (1.30, 1.70)	1.50 (1.30, 1.70)	1.50 (1.30, 1.80)	0.50
LDL (mmol/L)	2.70 (2.50, 2.90)	2.70 (2.50, 2.90)	2.70 (2.50, 2.90)	0.48
HDL (mmol/L)	1.20 (1.20, 1.30)	1.20 (1.20, 1.30)	1.20 (1.20, 1.30)	0.78
AST (U/L)	23.70 (21.00, 27.80)	23.70 (21.00, 27.80)	23.70 (21.00, 27.80)	0.66
ALT (U/L)	22.60 (19.10, 26.70)	22.60 (19.10, 26.80)	22.60 (19.20, 26.70)	0.58
TBIL (μmol/L)	14.80 (12.80, 17.10)	14.70 (12.80, 17.10)	14.80 (12.80, 17.00)	0.72
ALB (g/L)	41.20 (39.60, 42.40)	41.20 (39.60, 42.40)	41.20 (39.60, 42.40)	0.64
BUN (mmol/L)	6.20 (5.60, 7.00)	6.20 (5.60, 7.00)	6.20 (5.60, 7.00)	0.09
Scr (μmol/L)	75.50 (66.60, 86.60)	75.90 (66.70, 86.80)	75.20 (66.60, 86.50)	0.25
UA (μmol/L)	335.30 (305.30, 373.90)	334.90 (304.70, 373.40)	335.40 (305.40, 374.10)	0.62
PT (s)	13.60 (13.30, 14.00)	13.60 (13.30, 14.00)	13.60 (13.30, 14.00)	0.16
APTT (s)	35.30 (34.20, 36.70)	35.40 (34.20, 36.80)	35.30 (34.20, 36.70)	0.74
TT (s)	16.40 (16.10, 16.70)	16.40 (16.10, 16.70)	16.40 (16.10, 16.70)	0.34
INR	1.00 (1.00, 1.10)	1.00 (1.00, 1.10)	1.00 (1.00, 1.10)	0.63
D-dimer (mg/L)	0.92 (0.65, 1.49)	0.92 (0.66, 1.51)	0.92 (0.65, 1.49)	0.37
FIB (g/L)	3.60 (3.30, 3.90)	3.60 (3.30, 3.90)	3.60 (3.30, 3.90)	0.08
CK (U/L)	130.20 (103.40, 196.50)	131.00 (103.40, 202.00)	129.90 (103.30, 194.70)	0.22
CKMB (U/L)	12.60 (11.30, 15.20)	12.60 (11.20, 15.30)	12.60 (11.30, 15.20)	0.57
LDH (U/L)	203.60 (186.20, 224.20)	204.20 (186.40, 223.90)	203.40 (186.00, 224.30)	0.51
HBDH (U/L)	170.00 (154.60, 185.50)	170.00 (154.60, 185.60)	170.00 (154.60, 185.50)	0.86
IMA (U/L)	74.50 (71.00, 78.80)	74.70 (71.00, 78.90)	74.50 (71.00, 78.80)	0.28
Na (mmol/L)	138.70 (137.80, 139.60)	138.70 (137.70, 139.60)	138.70 (137.80, 139.60)	0.50
K (mmol/L)	3.79 (3.70, 3.87)	3.79 (3.70, 3.88)	3.79 (3.70, 3.87)	0.30
Cl (mmol/L)	103.50 (102.40, 104.50)	103.50 (102.40, 104.50)	103.50 (102.40, 104.50)	0.64
Ca (mmol/L)	2.20 (2.20, 2.20)	2.20 (2.20, 2.20)	2.20 (2.20, 2.20)	0.31
P (mmol/L)	0.99 (0.94, 1.03)	0.99 (0.94, 1.03)	0.99 (0.94, 1.04)	0.99
Lac (mmol/L)	2.50 (2.30, 2.70)	2.50 (2.30, 2.70)	2.50 (2.30, 2.70)	0.25
AG (mmol/L)	12.30 (11.58, 13.08)	12.30 (11.57, 13.09)	12.30 (11.59, 13.07)	0.72
TCO2(mmol/L)	22.70 (22.10, 23.50)	22.70 (22.10, 23.50)	22.70 (22.10, 23.50)	0.86

Data are presented as median (Q1, Q3) or *n* (%). Q1, 1st Quartile; Q3, 3rd Quartile; NIHSS, National Institutes of Health Stroke Scale; DVT, deep vein thrombosis; Subcortical, subcortical infarction; Posterior, posterior circulation infarction; LAA, large artery atherosclerosis; FLD, fatty liver disease; DM, diabetes mellitus; HTN, hypertension; CAD, coronary artery disease; AF, atrial fibrillation; Herniation, cerebral herniation; Hydro, hydrocephalus; HUA, hyperuricemia; HLP, hyperlipidemia; HypoAlb, hypoproteinemia; PLT, platelet count; WBC, white blood cell count; RBC, red blood cell count; HbA1c, hemoglobin A1c; CRP, C-reactive protein; TG, triglycerides; LDL, low-density lipoprotein cholesterol; HDL, high-density lipoprotein cholesterol; AST, aspartate aminotransferase; ALT, alanine aminotransferase; TBIL, total bilirubin; ALB, albumin; BUN, blood urea nitrogen; Scr, serum creatinine; UA, uric acid; PT, prothrombin time; APTT, activated partial thromboplastin time; TT, thrombin time; INR, international normalized ratio; FIB, fibrinogen; CK, creatine kinase; CKMB, creatine kinase-MB; LDH, lactate dehydrogenase; HBDH, α-hydroxybutyrate dehydrogenase; IMA, ischemia-modified albumin; Na, sodium; K, potassium; Cl, chloride; Ca, calcium; P, phosphorus; Lac, lactate; AG, anion gap; TCO_2_, total carbon dioxide.

### Feature selection

**Figure 1:**
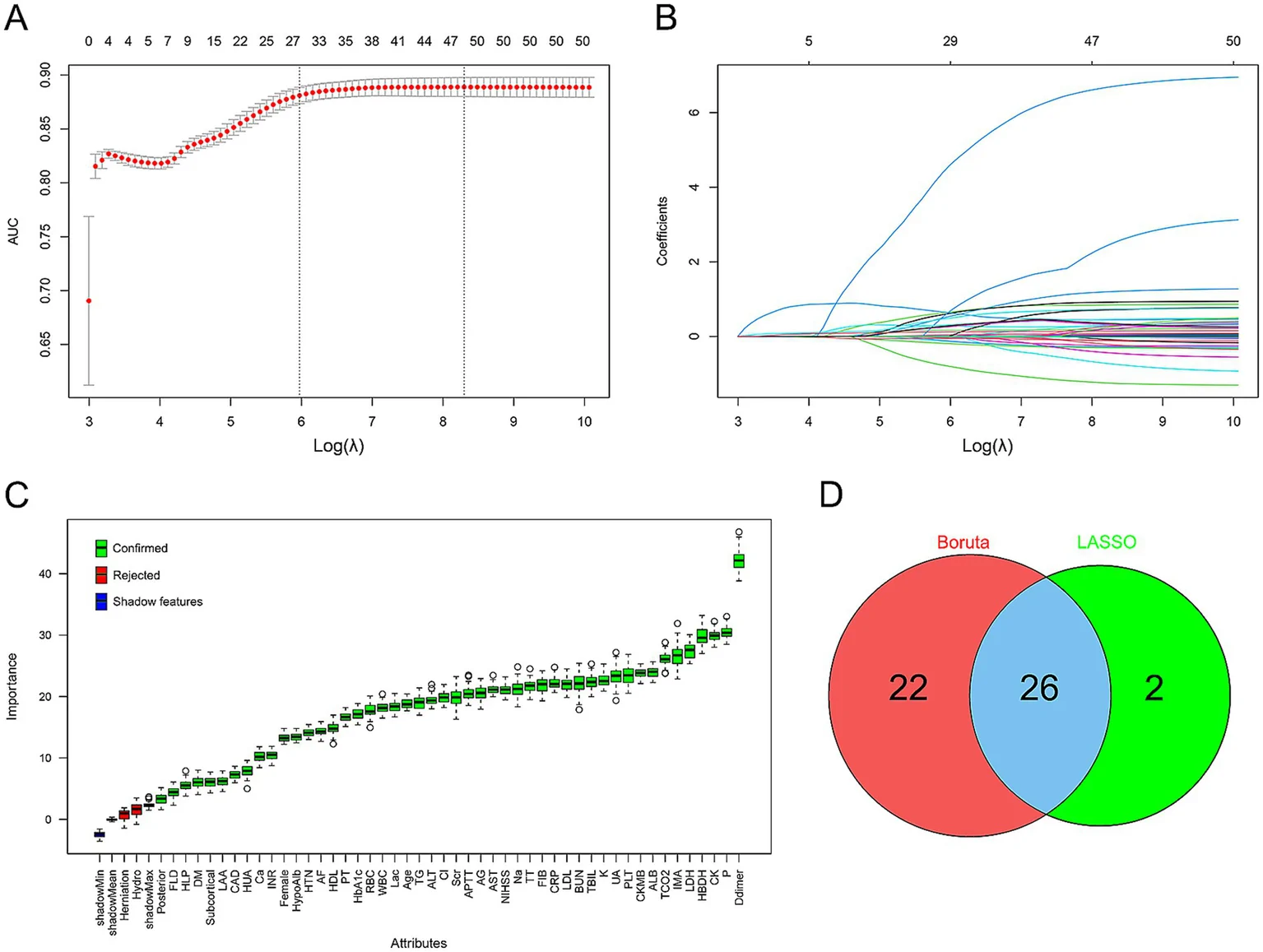
Variable selection using LASSO and Boruta. **(A)** Cross-validated performance of LASSO. **(B)** LASSO coefficient paths across the penalty grid. **(C)** Boruta feature importance. **(D)** Overlap of predictors retained by Boruta and LASSO.

Feature selection was performed using LASSO regression and the Boruta algorithm. LASSO selected predictors according to cross-validated model performance, whereas Boruta identified relevant variables based on permutation importance (Figures 1A–C). The union of predictors retained by the two methods included 26 variables (Figure 1D).

Multicollinearity was subsequently assessed using VIFs. Creatine kinase-MB showed evidence of severe multicollinearity, with a VIF greater than 10 (Table 2). After considering multicollinearity, predictor redundancy, clinical interpretability, predictive contribution, and routine availability, 19 variables were retained in the full predictor set: hypertension, atrial fibrillation, age, C-reactive protein, triglycerides, serum albumin, blood urea, activated partial thromboplastin time, thrombin time, international normalized ratio, D-dimer, α-hydroxybutyrate dehydrogenase, ischemia-modified albumin, serum chloride, serum phosphorus, lactate, anion gap, total carbon dioxide, and NIHSS score.

**Table 2:** Assessment of multicollinearity using variance inflation factors.

Variables	Variance inflation factor
Hypertension	1.399
Atrial fibrillation	1.321
Hypoproteinemia	2.798
Age	2.019
White blood cell count	2.761
Red blood cell count	2.084
C-reactive protein	5.357
Triglycerides	1.778
Low density lipoprotein cholesterol	1.550
Alanine aminotransferase	1.650
Serum albumin	3.957
Blood urea	1.754
Activated partial thromboplastin time	1.697
Thrombin time	1.313
International normalized ratio	1.654
D-dimer	4.320
Creatine kinase	8.574
Creatine kinase-MB	12.212
α-hydroxybutyrate dehydrogenase	5.871
Ischemia-modified albumin	1.388
Serum chloride	1.627
Serum phosphorus	1.708
Lactate	1.619
Anion gap	2.914
Total carbon dioxide	2.624
National Institutes of Health Stroke Scale Score	1.803

### Full predictor-set model performance

Using the 19-variable full predictor set, we trained and evaluated 8 machine learning algorithms. Most models demonstrated favorable discrimination. RANGER achieved the highest AUC in the held-out test set (0.976; 95% CI, 0.971–0.981) (Supplementary Figures S1A,B). Repeated 5-fold cross-validation with 2 repeats showed consistently high ROC AUCs with limited variation across resampling iterations (Supplementary Figure S1C). Calibration curves showed generally acceptable agreement between predicted and observed risks, and decision curve analysis suggested potential net clinical benefit for most models across a range of threshold probabilities (Supplementary Figures S2A–D). SHAP analysis of the RANGER model identified D-dimer as the most influential predictor (Supplementary Figures S3A,B).

### Simplified predictor-set model performance

Based on SHAP-derived importance and considerations of clinical availability, interpretability, measurement burden, and implementation feasibility, eight variables were retained in the simplified predictor set: age, atrial fibrillation, D-dimer, NIHSS score, C-reactive protein, serum albumin, lactate, and blood urea. All eight machine learning algorithms were retrained using this predictor set.

**Figure 2:**
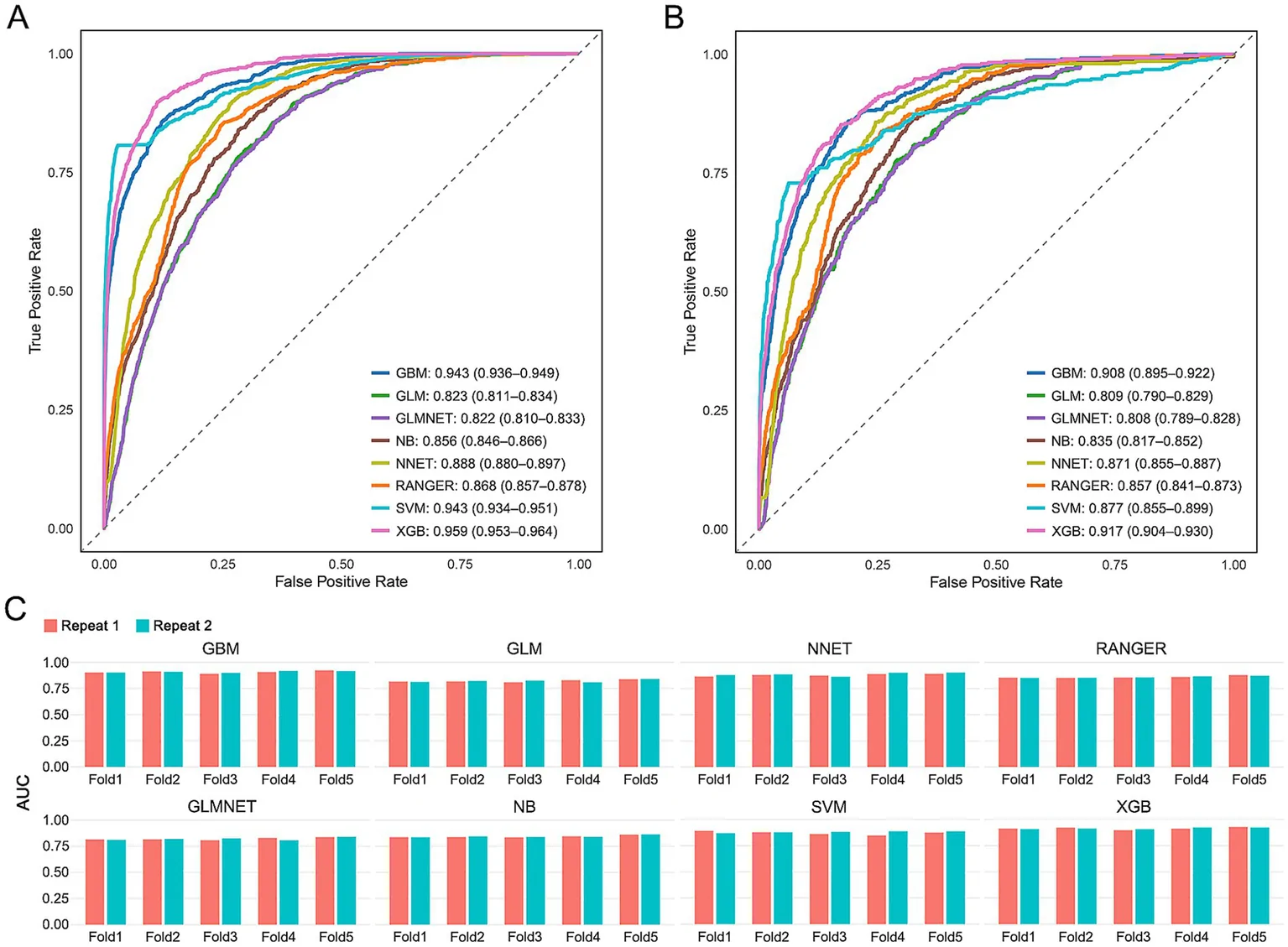
Discrimination and cross-validation performance of the simplified predictor-set model. **(A)** Receiver operating characteristic (ROC) curves of the simplified model in the training set. **(B)** ROC curves of the simplified model in the held-out test set. **(C)** Area under the ROC curve (AUC) across repeated 5-fold cross-validation (2 repeats) for the simplified model.

**Figure 3:**
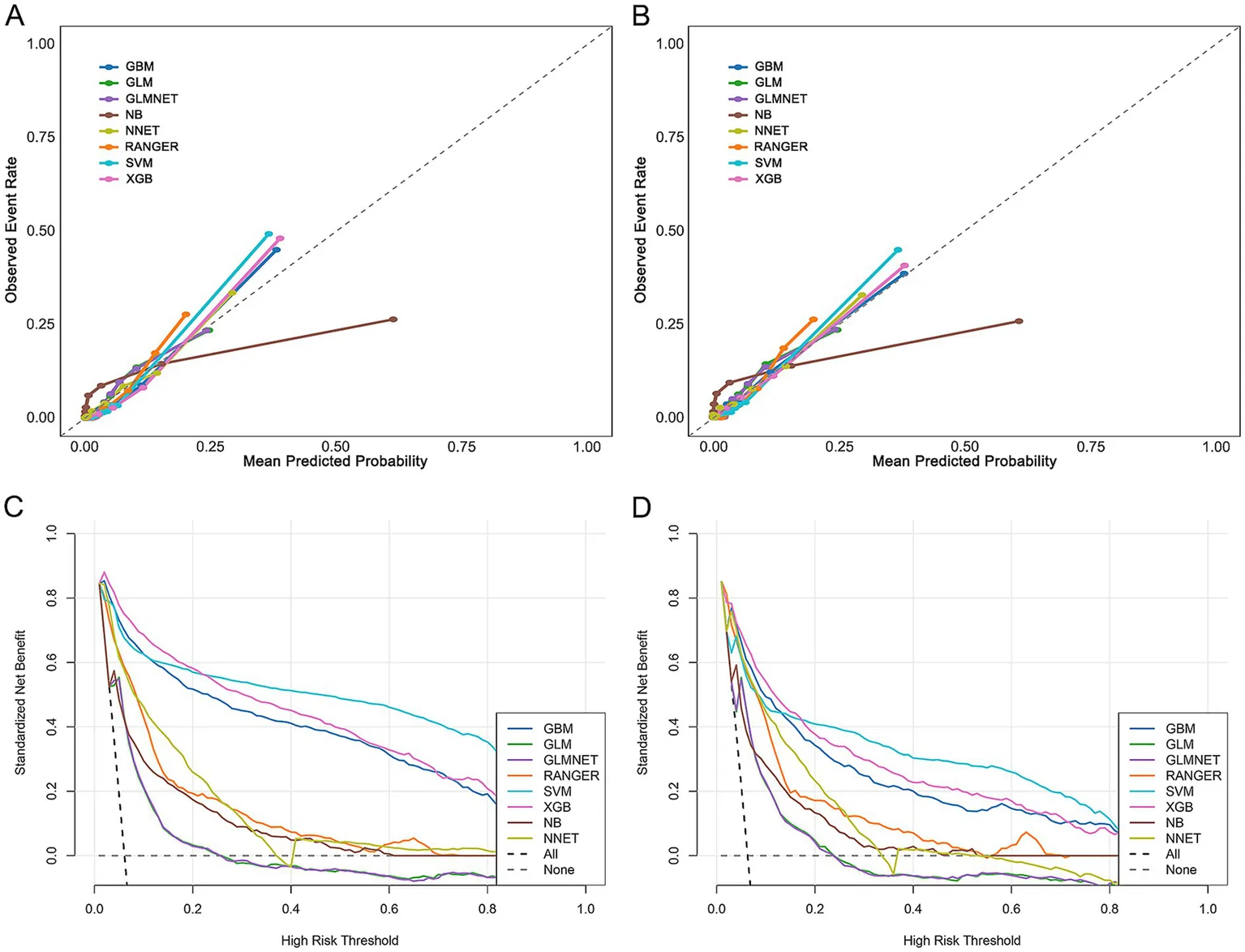
Calibration and decision curve analyses of the simplified predictor-set models. **(A)** Calibration curve in the training set. **(B)** Calibration curve in the held-out test set. **(C)** Decision curve analysis in the training set. **(D)** Decision curve analysis in the held-out test set.

XGBoost achieved the highest ROC AUC among the simplified predictor-set models, with values of 0.959 (95% CI, 0.953–0.964) in the training set and 0.917 (95% CI, 0.904–0.930) in the held-out test set (Figures 2A,B). However, because of the low incidence of DVT, model comparison placed greater emphasis on metrics informative for imbalanced outcomes. In the held-out test set, SVM achieved the highest AUPRC (0.605), the lowest Brier score (0.038), the highest positive predictive value (0.440), and the highest *F*1-score (0.549), demonstrating the most favorable overall performance profile. XGBoost achieved the highest sensitivity (0.852), reflecting a different trade-off in which case detection was prioritized over positive predictive value (Table 3). Repeated 5-fold cross-validation with 2 repeats showed generally consistent ROC AUCs across resampling iterations (Figure 2C). Calibration and decision curve analyses suggested acceptable predictive agreement and potential net clinical benefit for several models (Figures 3A–D).

**Table 3:** Performance metrics of machine learning models in the train and test sets.

Model	Split	AUPRC	Brier	Threshold	Accuracy	Sensitivity	Specificity	PPV	NPV	*F*1
GBM	Train	0.686	0.033	0.059	0.815	0.900	0.809	0.235	0.992	0.373
	Test	0.530	0.041	0.059	0.815	0.860	0.812	0.235	0.988	0.369
GLM	Train	0.197	0.054	0.054	0.717	0.786	0.712	0.151	0.981	0.253
	Test	0.193	0.056	0.054	0.715	0.776	0.711	0.153	0.979	0.256
GLMNET	Train	0.197	0.054	0.050	0.682	0.817	0.673	0.140	0.983	0.239
	Test	0.192	0.056	0.050	0.685	0.803	0.677	0.143	0.981	0.243
RANGER	Train	0.360	0.049	0.064	0.751	0.856	0.744	0.179	0.988	0.296
	Test	0.339	0.051	0.064	0.748	0.837	0.742	0.180	0.985	0.296
SVM	Train	0.782	0.027	0.069	0.961	0.806	0.971	0.646	0.987	0.717
	Test	0.605	0.038	0.069	0.924	0.729	0.937	0.440	0.981	0.549
XGB	Train	0.730	0.031	0.068	0.836	0.931	0.830	0.263	0.995	0.410
	Test	0.562	0.040	0.068	0.829	0.852	0.827	0.249	0.988	0.386
NB	Train	0.315	0.063	0.006	0.690	0.875	0.678	0.150	0.988	0.256
	Test	0.265	0.067	0.006	0.688	0.865	0.676	0.152	0.987	0.259
NNET	Train	0.350	0.046	0.066	0.767	0.862	0.761	0.190	0.988	0.312
	Test	0.301	0.049	0.066	0.761	0.857	0.754	0.190	0.987	0.311

AUPRC, area under the precision–recall curve; PPV, positive predictive value; NPV, negative predictive value; GBM, gradient boosting machine; GLM, generalized linear model (logistic regression); GLMNET, generalized linear model with elastic-net regularization; RANGER, random forest (ranger implementation); SVM, support vector machine; XGB, extreme gradient boosting; NB, naive Bayes; NNET, feed-forward neural network.

**Figure 4:**
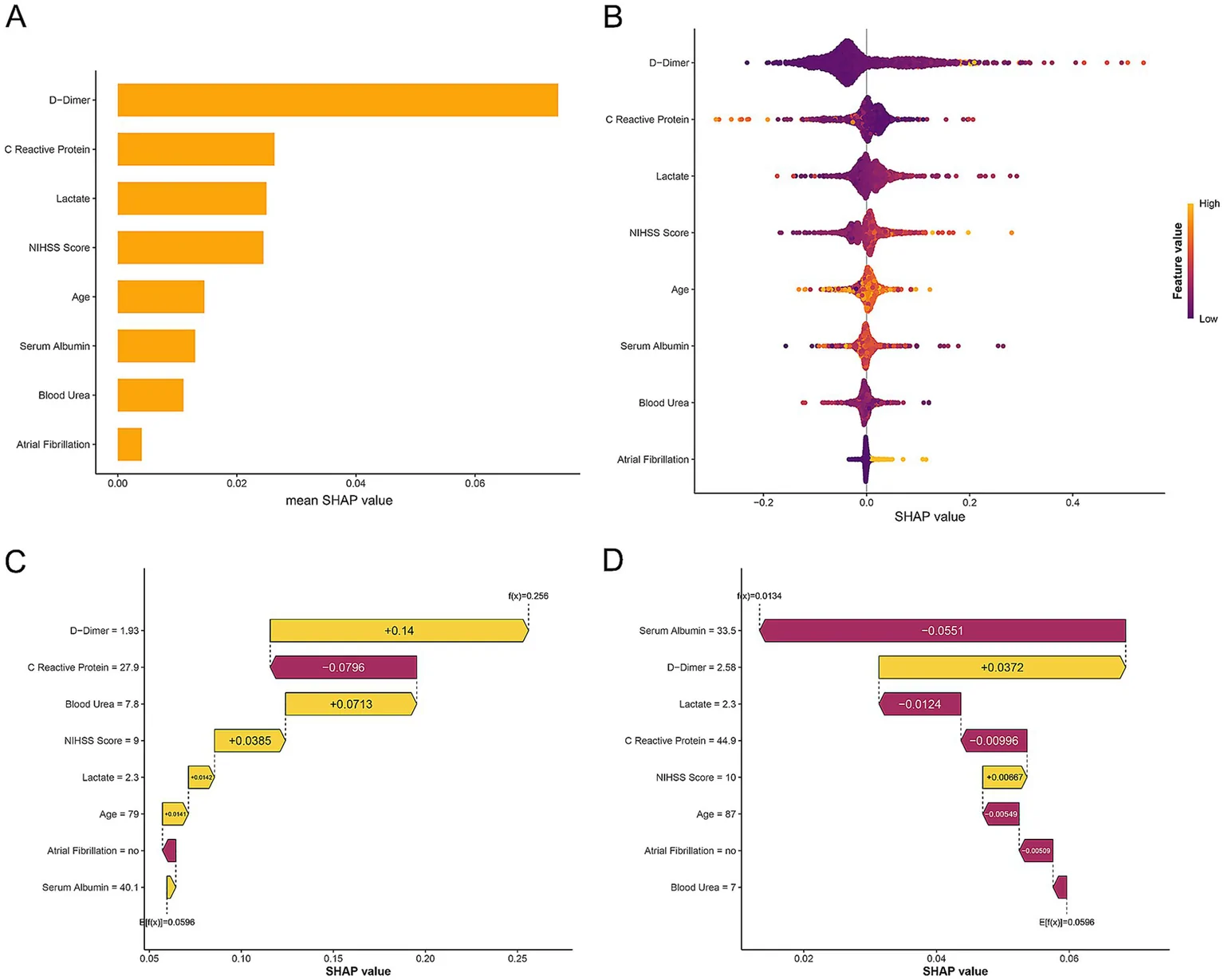
SHAP-based interpretation of the simplified XGBoost model. **(A)** SHAP feature importance plot showing the relative contribution of each predictor. **(B)** SHAP summary plot illustrating the direction and magnitude of feature effects. **(C,D)** SHAP force plots for 2 representative patients, illustrating how individual features contributed to model predictions.

**Figure 5:**
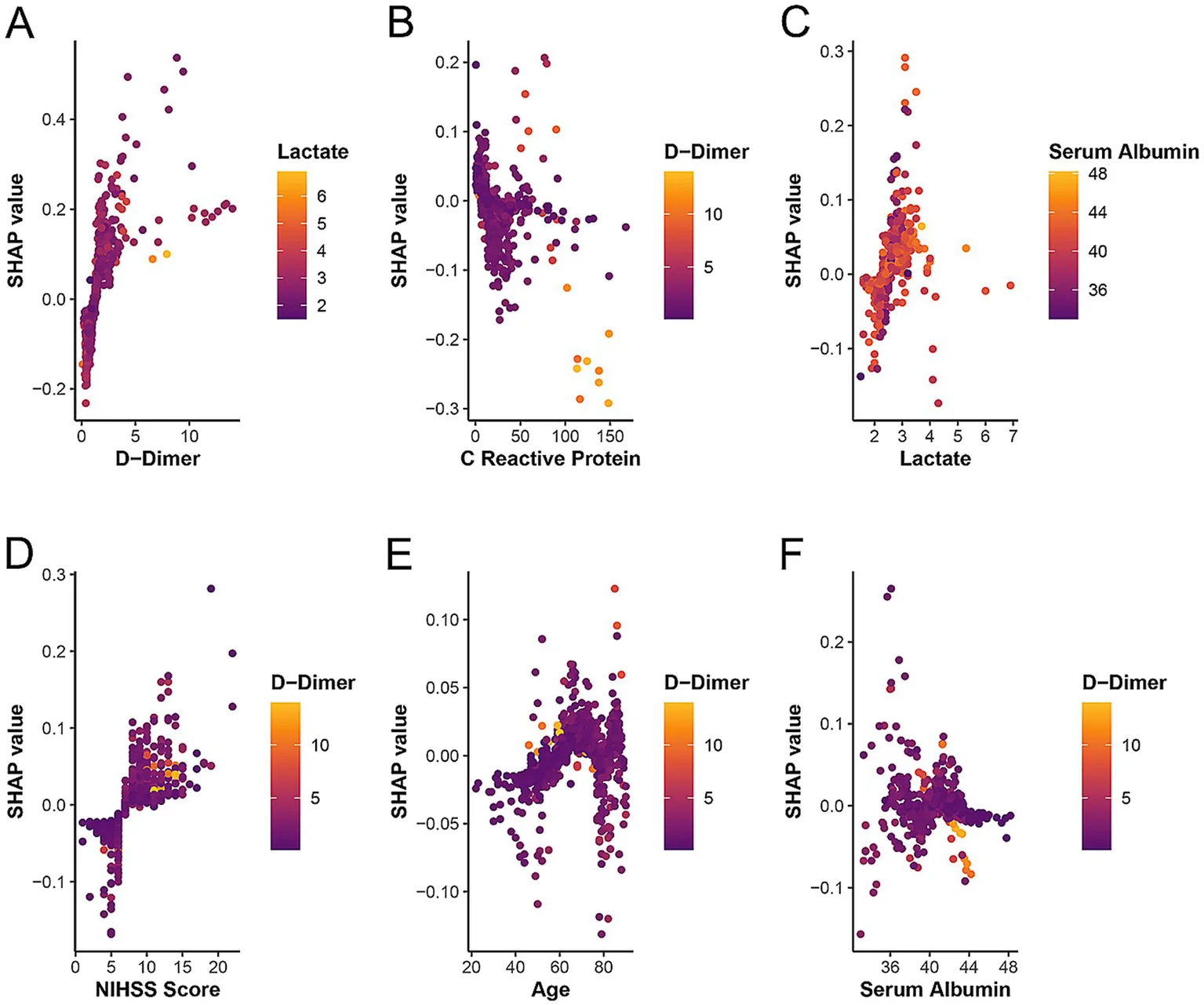
SHAP dependence plots for key predictors in the simplified XGBoost model. **(A–F)** SHAP dependence plots showing the associations of key predictors with the predicted risk of deep vein thrombosis. Each panel illustrates how variation in an individual predictor influenced the model output.

XGBoost was selected for detailed SHAP visualization as a representative nonlinear tree-based model and not because it was the overall best-performing algorithm. D-dimer was the most influential predictor in the simplified XGBoost model, followed by C-reactive protein, lactate, and NIHSS score (Figures 4A,B). SHAP force plots illustrated patient-level feature contributions in 2 representative cases (Figures 4C,D). SHAP dependence plots showed that higher D-dimer and C-reactive protein levels were associated with higher predicted DVT risk, whereas higher serum albumin levels tended to be associated with lower predicted risk (Figures 5A–F).

**Figure 6:**
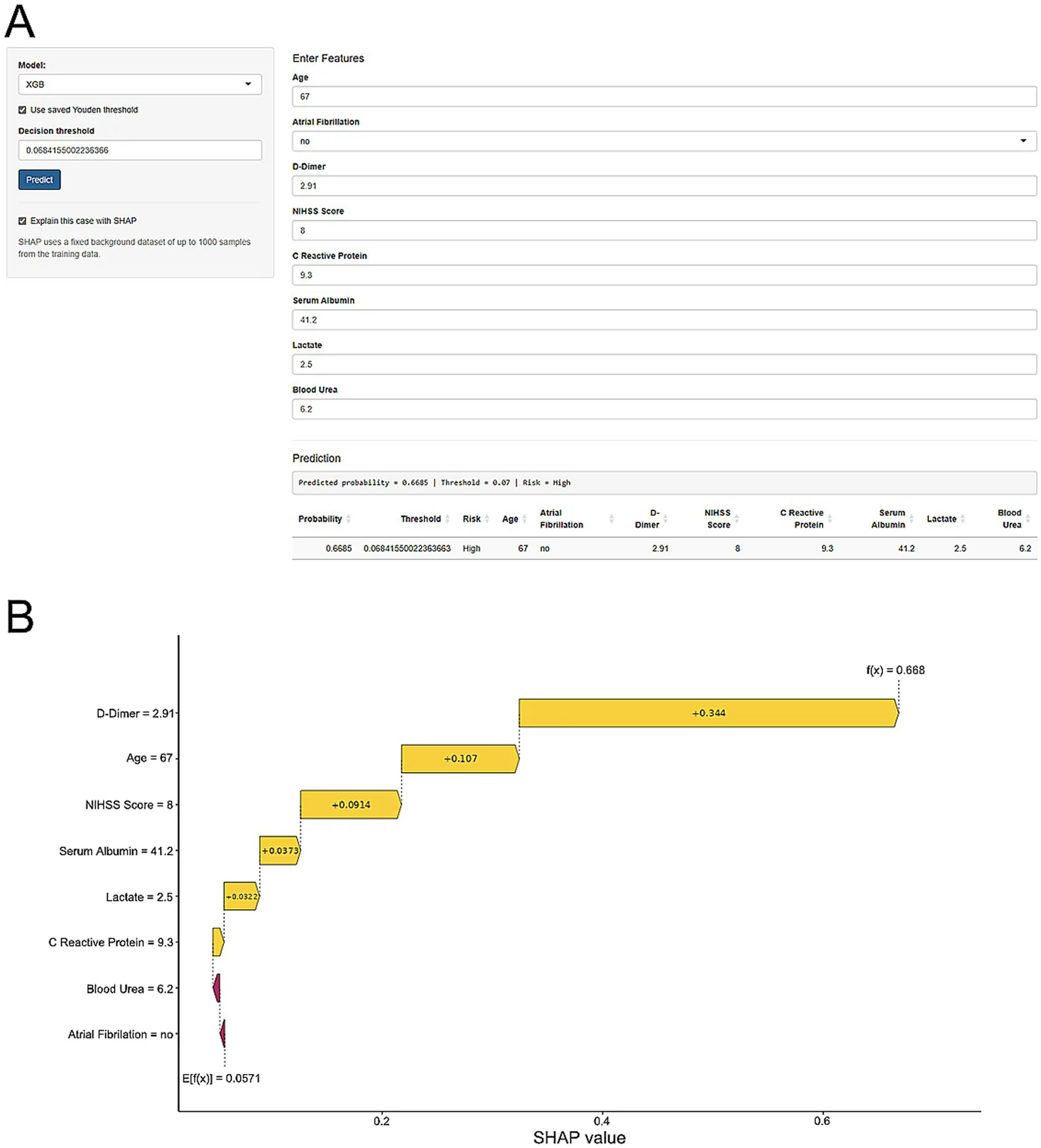
Online prediction platform incorporating 8 machine learning algorithms using the simplified predictor set. **(A)** Input interface of the online calculator showing the 8 predictors included in the simplified model. **(B)** Output interface of the online calculator showing individualized risk prediction and feature contribution visualization.

All eight algorithms trained using the simplified predictor set were incorporated into the online prediction platform, allowing users to select an algorithm and obtain the corresponding individualized risk estimate and available explanatory output (Figures 6A,B).

### Sensitivity analyses

After D-dimer was excluded from the full predictor set, all 8 algorithms were retrained using the remaining 18 variables. Model discrimination was attenuated compared with the primary full predictor-set analysis, as reflected by lower ROC AUCs across models (Supplementary Figures S4A–C). Nevertheless, calibration and decision curve analyses indicated that several models retained acceptable calibration and potential net clinical benefit (Supplementary Figures S5A–D). SHAP analysis of the XGBoost model identified α-hydroxybutyrate dehydrogenase as the most influential predictor after exclusion of D-dimer (Supplementary Figures S6A,B).

After D-dimer was excluded from the simplified predictor set, all 8 algorithms were retrained using the remaining 7 variables. Discrimination was also attenuated compared with the primary simplified predictor-set analysis (Supplementary Figures S7A–C). Calibration and decision curve analyses indicated that the models retained moderate predictive performance and potential net clinical benefit (Supplementary Figures S8A–D). SHAP analysis of the XGBoost model identified lactate as the most influential predictor (Supplementary Figures S9A,B).

In the SMOTE sensitivity analysis using the simplified 8-variable predictor set, model discrimination decreased slightly compared with the primary analysis (Supplementary Figures S10A–C). Calibration plots showed greater discrepancies between predicted and observed risks, although decision curve analysis continued to suggest potential net clinical benefit across a range of threshold probabilities (Supplementary Figures S11A–D). SHAP analysis of the XGBoost model identified D-dimer and NIHSS score as the most influential predictors (Supplementary Figures S12A,B).

## Discussion

In this multicenter secondary analysis of a large cohort of patients with AIS, we developed and internally evaluated eight machine learning algorithms using both full and simplified predictor sets for the prediction of in-hospital DVT. Among the full predictor-set models, RANGER achieved the highest AUC. After predictor reduction, SVM demonstrated the most favorable overall performance profile, with the highest AUPRC and positive predictive value, the lowest Brier score, and the highest *F*1-score in the held-out test set. XGBoost achieved the highest AUC and sensitivity, reflecting a different trade-off between case detection and false-positive predictions. All eight algorithms trained using the simplified predictor set were incorporated into the online prediction platform rather than restricting implementation to a single model. Sensitivity analyses further showed that model performance was attenuated after exclusion of D-dimer and decreased slightly after within-fold SMOTE, underscoring the importance of evaluating model performance using multiple complementary metrics.

The incidence of in-hospital DVT in the present cohort was 6.17%, which was lower than the previously reported incidence of 8.9 to 28% among patients with acute stroke in China (7). This difference may partly reflect variation in patient characteristics and DVT ascertainment because DVT was identified through routine clinical evaluation with imaging confirmation rather than systematic screening of all patients. Consequently, asymptomatic or clinically unsuspected DVT may have been underdetected.

Previous poststroke DVT prediction studies have mainly relied on conventional clinical scores or logistic regression–based nomograms. In a multicenter prospective study, Liu et al. (19) developed a 7-point clinical score based on age, sex, obesity, active cancer, stroke subtype, and lower-limb weakness, with c statistics of 0.70 in the derivation cohort and 0.65 in the overall cohort. Subsequent studies developed nomograms for more specific populations or clinical settings. Wang et al. (20) reported AUCs of 0.833 and 0.907 for a 6-variable nomogram among patients with AIS receiving thrombolytic therapy, whereas Chen et al. (21) reported AUCs of 0.812 and 0.796 in modeling and external validation cohorts, respectively. Xu and Yin (22) developed a 5-variable nomogram in a single-center cohort of 369 patients. More recently, Jiang et al. (23) applied machine learning to predict venous thromboembolism in 1632 patients with AIS, and their gradient boosting model achieved an AUC of 0.923. However, a 2024 systematic review and meta-analysis found that most published acute stroke DVT prediction models were based on logistic regression, involved relatively modest sample sizes, and were judged to have a high risk of bias; the pooled AUC of validated models was 0.76 (7). The present study extends this literature in several respects. First, the analysis included 21,459 patients from 4 hospitals, providing a substantially larger sample than most previous studies. Second, rather than evaluating a single modeling approach, we systematically compared eight algorithms using the same data partitioning and internal evaluation framework. Third, model comparison incorporated AUPRC, positive predictive value, sensitivity, *F*1-score, Brier score, calibration, and decision curve analysis in addition to ROC AUC, which was particularly important given the low incidence of DVT. Fourth, all 8 algorithms were retrained using a simplified 8-variable predictor set and incorporated into an online platform, allowing their differing performance profiles to be explored. Nevertheless, the high ROC AUC observed for the full RANGER model should be interpreted cautiously because evaluation was limited to a held-out test set derived from the same source cohort.

The predictors retained in the simplified predictor set were clinically and biologically plausible. D-dimer, which was the most influential predictor in both the full and simplified models, reflects activation of coagulation and fibrinolysis and has been consistently associated with venous thromboembolism, including among patients with ischemic stroke (24, 25). Model discrimination decreased after D-dimer was excluded, suggesting that this biomarker contributed substantially to predictive performance. Nevertheless, the remaining clinical and laboratory variables retained predictive information, indicating that model performance was not entirely dependent on D-dimer. After exclusion of D-dimer, α-hydroxybutyrate dehydrogenase and lactate emerged as influential predictors in the full and simplified predictor-set analyses, respectively.

Elevated C-reactive protein may reflect thromboinflammation and endothelial activation, which may contribute to thrombosis (26–28). Higher serum albumin was associated with lower predicted risk, consistent with evidence linking hypoalbuminemia to an increased risk of venous thromboembolism and potentially reflecting adverse inflammatory or nutritional status (29). Blood urea may reflect dehydration-related metabolic abnormalities that have previously been associated with venous thromboembolism after ischemic stroke (30). Age, atrial fibrillation, and NIHSS score may capture patient vulnerability, stroke severity, and immobility burden, which are established contributors to DVT risk (31–34). These SHAP-based findings should be interpreted as descriptions of model behavior rather than evidence of causal associations.

The SMOTE sensitivity analysis provided additional information regarding class imbalance. Although within-fold SMOTE was intended to increase representation of patients with DVT during model training, discrimination decreased slightly and discrepancies between predicted and observed risks increased. Decision curve analysis nevertheless suggested potential net benefit across selected threshold probabilities. These findings illustrate that oversampling does not necessarily improve overall model performance and may alter the balance among sensitivity, positive predictive value, and calibration. They also support the use of AUPRC, *F*1-score, and calibration measures alongside ROC AUC when evaluating models for infrequent outcomes.

This study has several strengths. It used a large multicenter dataset, compared 8 machine learning algorithms under a common evaluation framework, and assessed discrimination, calibration, class imbalance–sensitive metrics, and potential clinical utility. Predictor reduction considered SHAP-derived importance, clinical availability, interpretability, measurement burden, and implementation feasibility. Sensitivity analyses excluding D-dimer and using within-fold SMOTE further evaluated the robustness of the findings. In addition, deployment of all 8 simplified predictor-set models in an online platform provides a basis for future evaluation of model usability and transportability.

Several limitations should be acknowledged. First, this was a retrospective secondary analysis of a dataset originally assembled for a different poststroke research purpose. The source cohort eligibility criteria and available variables may therefore not represent an unselected AIS population, which may have introduced selection bias. Second, the publicly released dataset included only complete observations. We could not assess the amount, pattern, or mechanism of missingness or compare included patients with those excluded because of incomplete data. Third, detailed information regarding preadmission antiplatelet or anticoagulant therapy, in-hospital pharmacologic or mechanical thromboprophylaxis, and changes in antithrombotic treatment was unavailable. These treatment-related factors may have affected the occurrence of DVT and the transportability of the models. Fourth, information on postadmission complications, including hemorrhagic transformation, acute coronary syndrome, and other major clinical events, was unavailable. Although these events would not generally be appropriate baseline predictors, they may have influenced subsequent treatment, mobility, and DVT risk. Fifth, although candidate predictors were described as baseline or admission measurements in the source dataset, exact timestamps relative to DVT diagnosis were unavailable. Temporal ambiguity and diagnostic-process–related information leakage therefore cannot be completely excluded. Sixth, DVT was identified according to routine clinical practice rather than systematic screening, and asymptomatic events may have been missed. Seventh, all participating hospitals were located in Chongqing, China, and nationality and ethnicity were not recorded, limiting assessment of transportability across geographic, ethnic, and health care settings. Finally, the held-out test set was derived from the same source cohort and therefore provided internal rather than external validation. An independent cohort with compatible predictor definitions and DVT ascertainment was not available. Consequently, the models and online platform should be regarded as research tools rather than instruments ready for routine clinical decision-making. Independent external validation, prospective calibration assessment, and clinical-impact studies are required before model-guided thromboprophylaxis can be recommended.

## Conclusion

In this multicenter study of patients with acute ischemic stroke, machine learning models demonstrated favorable performance for predicting in-hospital deep vein thrombosis. Among models using the simplified 8-variable predictor set, SVM showed the most favorable overall performance, whereas XGBoost achieved the highest sensitivity. All 8 algorithms were incorporated into an online prediction platform. Independent external validation and prospective clinical-impact assessment are required before routine clinical implementation.

## Data Availability

Publicly available datasets were analyzed in this study. This data can be found here: The data used in this secondary analysis are publicly available in the Dryad Digital Repository (DOI: 10.5061/dryad.w0vt4b92c).

## Supplementary material

SUPPLEMENTARY FIGURE S1 Discrimination and cross-validation performance of the full model. (A) ROC curves of the full model in the training set. (B) ROC curves of the full model in the held-out test set. C, AUC across repeated 5-fold cross-validation (2 repeats) for the full model.

SUPPLEMENTARY FIGURE S2 Calibration and clinical utility of the full model. (A) Calibration curves in the training set. (B) Calibration curves in the held-out test set. (C) Decision curve analysis in the training set. (D) Decision curve analysis in the test set.

SUPPLEMENTARY FIGURE S3 SHAP-based interpretation of the full RANGER model. (A) SHAP feature importance plot for the full RANGER model. (B) SHAP summary plot showing the contribution of individual variables to model predictions.

SUPPLEMENTARY FIGURE S4 Discrimination and cross-validation performance of the full predictor-set models after exclusion of D-dimer. (A) Receiver operating characteristic curves for the 8 machine learning algorithms in the training set. (B) Receiver operating characteristic curves for the 8 machine learning algorithms in the held-out test set. (C) ROC AUCs across repeated 5-fold cross-validation with 2 repeats. All models were trained using the remaining 18 predictors after exclusion of D-dimer.

SUPPLEMENTARY FIGURE S5 Calibration and decision curve analyses of the full predictor-set models after exclusion of D-dimer. (A) Calibration curves in the training set. (B) Calibration curves in the held-out test set. (C) Decision curve analysis in the training set. (D) Decision curve analysis in the held-out test set. All models were trained using the remaining 18 predictors after exclusion of D-dimer.

SUPPLEMENTARY FIGURE S6 SHAP-based interpretation of the full predictor-set XGBoost model after exclusion of D-dimer. (A) SHAP feature importance plot showing the relative contribution of each predictor. (B) SHAP summary plot illustrating the direction and magnitude of individual predictor contributions to model output. The model was trained using the remaining 18 predictors after exclusion of D-dimer.

SUPPLEMENTARY FIGURE S7 Discrimination and cross-validation performance of the simplified predictor-set models after exclusion of D-dimer. (A) Receiver operating characteristic curves for the 8 machine learning algorithms in the training set. (B) Receiver operating characteristic curves for the 8 machine learning algorithms in the held-out test set. (C) ROC AUCs across repeated 5-fold cross-validation with 2 repeats. All models were trained using the remaining 7 predictors after exclusion of D-dimer.

SUPPLEMENTARY FIGURE S8 Calibration and decision curve analyses of the simplified predictor-set models after exclusion of D-dimer. (A) Calibration curves in the training set. (B) Calibration curves in the held-out test set. (C) Decision curve analysis in the training set. (D) Decision curve analysis in the held-out test set. All models were trained using the remaining 7 predictors after exclusion of D-dimer.

SUPPLEMENTARY FIGURE S9 SHAP-based interpretation of the simplified predictor-set RANGER model after exclusion of D-dimer. (A) SHAP feature importance plot showing the relative contribution of each predictor. (B) SHAP summary plot illustrating the direction and magnitude of individual predictor contributions to model output. The model was trained using the remaining 7 predictors after exclusion of D-dimer.

SUPPLEMENTARY FIGURE S10 Discrimination and cross-validation performance of the simplified predictor-set models in the SMOTE sensitivity analysis. (A) Receiver operating characteristic curves for the 8 machine learning algorithms in the training set. (B) Receiver operating characteristic curves for the 8 machine learning algorithms in the held-out test set. (C) ROC AUCs across repeated 5-fold cross-validation with 2 repeats. SMOTE was applied exclusively within the training portion of each cross-validation iteration, whereas the corresponding assessment folds and the held-out test set retained the original DVT prevalence.

SUPPLEMENTARY FIGURE S11 Calibration and decision curve analyses of the simplified predictor-set models in the SMOTE sensitivity analysis. (A) Calibration curves in the training set. (B) Calibration curves in the held-out test set. (C) Decision curve analysis in the training set. (D) Decision curve analysis in the held-out test set. SMOTE was applied exclusively within the training portion of each cross-validation iteration; the held-out test set was not resampled.

SUPPLEMENTARY FIGURE S12 SHAP-based interpretation of the simplified predictor-set RANGER model in the SMOTE sensitivity analysis. (A) SHAP feature importance plot showing the relative contribution of each predictor. (B) SHAP summary plot illustrating the direction and magnitude of individual predictor contributions to model output. The model was trained using the simplified 8-variable predictor set with within-fold SMOTE.
