# Supplementary material for: Machine learning–based prediction of in-hospital deep vein thrombosis in patients with acute ischemic stroke: a multicenter study
Source: Front Neurol. 2026 Aug 14;17:1847449. doi: 10.3389/fneur.2026.1847449 (PMC13521839; doi:10.3389/fneur.2026.1847449)
Supplement: Supplementary file 1 [file Table_1.DOCX]

Table S1. Model-specific implementation, preprocessing procedures, and candidate hyperparameter search spaces

| **Algorithm** | **R implementation** | **Preprocessing** | **Candidate hyperparameters** | **Fixed settings** |
| --- | --- | --- | --- | --- |
| GBM | caret, method="gbm"; gbm package | Removal of zero- and near-zero-variance predictors | n.trees: 600, 1200; interaction.depth: 1, 2, 3; shrinkage: 0.01, 0.03, 0.05; n.minobsinnode: 20, 40, 60 | Bernoulli distribution |
| GLM | caret, method="glm"; stats package | Removal of zero- and near-zero-variance predictors | No hyperparameter tuning | Binomial family with logit link |
| GLMNET | caret, method="glmnet"; glmnet package | Centering, scaling, and removal of zero- and near-zero-variance predictors | alpha: 0, 0.5, 1; lambda: 0.001 to 1, with 7 logarithmically spaced values | Binomial model |
| RANGER | caret, method="ranger"; ranger package | Removal of zero- and near-zero-variance predictors | mtry: 1, 2; splitrule: gini; min.node.size: 20, 50, 100 | 300 trees; maximum depth, 3; permutation importance |
| SVM | caret; kernlab package | Centering, scaling, and removal of zero- and near-zero-variance predictors | Primary analysis: RBF kernel, sigma=0.208918 and C=0.25–16; SMOTE analysis: linear kernel, C=0.01, 0.1, 1 | Kernel differed in the SMOTE analysis because of computational burden |
| XGBoost | caret, method="xgbTree"; xgboost package | Removal of zero- and near-zero-variance predictors | nrounds: 300, 800; max_depth: 2, 3; eta: 0.02, 0.05; gamma: 1, 3; colsample_bytree: 0.6, 0.8; min_child_weight: 5, 10; subsample: 0.6, 0.8 | Binary probabilistic classification |
| Naive Bayes | caret, method="naive_bayes"; naivebayes package | Centering, scaling, and removal of zero- and near-zero-variance predictors | laplace: 0, 1; usekernel: No, Yes; adjust: 1, 1.5 | None |
| NNET | caret, method="nnet"; nnet package | Centering, scaling, and removal of zero- and near-zero-variance predictors | size: 2, 4, 6; decay: 0, 0.0001, 0.001 | Single hidden layer; maximum number of weights, 10,000 |

Hyperparameters were optimized by maximizing the mean ROC AUC during stratified repeated 5-fold cross-validation with 2 repeats in the training set. Zero- and near-zero-variance predictors were removed for all models. Centering and scaling were additionally applied for GLMNET, SVM, naive Bayes, and NNET. The same model-specific preprocessing procedures and candidate hyperparameter search spaces were used in the full predictor-set and D-dimer-exclusion analyses. In the SMOTE sensitivity analysis, a linear-kernel SVM was used to reduce computational burden.
