# Supplementary material for: Machine learning–based prediction of in-hospital deep vein thrombosis in patients with acute ischemic stroke: a multicenter study
Source: Front Neurol. 2026 Aug 14;17:1847449. doi: 10.3389/fneur.2026.1847449 (PMC13521839; doi:10.3389/fneur.2026.1847449)
Supplement: Supplementary file 2 [file Table_2.DOCX]

Table S2. Hyperparameters selected for the simplified predictor-set models in the primary and SMOTE sensitivity analyses

| **Algorithm** | **Primary analysis: simplified 8-variable predictor set** | **SMOTE sensitivity analysis: simplified 8-variable predictor set** |
| --- | --- | --- |
| GBM | n.trees=1200; interaction.depth=3; shrinkage=0.05; n.minobsinnode=60 | n.trees=1200; interaction.depth=3; shrinkage=0.05; n.minobsinnode=60 |
| GLM | Not applicable | Not applicable |
| GLMNET | alpha=0.5; lambda=0.001 | alpha=1; lambda=0.01 |
| RANGER | mtry=2; splitrule=gini; min.node.size=100 | mtry=2; splitrule=gini; min.node.size=100 |
| SVM | RBF kernel; sigma=0.208918; C=16 | Linear kernel; C=1 |
| XGBoost | nrounds=800; max_depth=3; eta=0.05; gamma=1; colsample_bytree=0.8; min_child_weight=5; subsample=0.6 | Same as primary analysis |
| Naive Bayes | laplace=0; usekernel=Yes; adjust=1 | laplace=0; usekernel=Yes; adjust=1 |
| NNET | size=6; decay=0.0001 | size=6; decay=0.001 |

Hyperparameters were selected according to the highest mean ROC AUC across the repeated cross-validation resamples. Logistic regression did not require hyperparameter tuning. SMOTE was performed exclusively within the analysis portion of each cross-validation iteration; the corresponding assessment folds and held-out test set retained the original DVT prevalence.
